# The VUS Challenge in Cystic Kidney Disease: A Case-Based Review

**DOI:** 10.34067/KID.0000000000000298

**Published:** 2023-11-14

**Authors:** Abinet M. Aklilu, Ashima Gulati, Kayla J. Kolber, Hana Yang, Peter C. Harris, Neera K. Dahl

**Affiliations:** 1 Section of Nephrology, Yale University School of Medicine, New Haven, Connecticut; 2 Children's National Hospital, Washington, DC; 3 Center for Individualized Medicine, Mayo Clinic, Rochester, Minnesota; 4 Division of Nephrology and Hypertension, Mayo Clinic, Rochester, Minnesota

**Keywords:** ADPKD, cystic kidney, human genetics

## Abstract

Genetic testing in nephrology is becoming increasingly important to diagnose patients and to provide appropriate care. This is especially true for autosomal dominant polycystic kidney disease (ADPKD) because this is a common cause of kidney failure and genetically complex. In addition to the major genes, *PKD1* and *PKD2*, there are at least six minor loci, and phenotypic, and in some cases, genetic overlap with other cystic disorders. Targeted next-generation sequencing, a low-cost, high-throughput technique, has made routine genetic testing viable in nephrology clinics. Appropriate pre- and post-testing genetic counseling is essential to the testing process. Carefully assessing variants is also critical, with the genetic report classifying variants in accordance with American College of Medical Genetics and Genomics guidelines. However, variant of uncertain significance (VUSs) may pose a significant challenge for the ordering clinician. In ADPKD, and particularly within *PKD1*, there is high allelic heterogeneity; no single variant is present in more than 2% of families. The Mayo/Polycystic Kidney Disease Foundation variant database, a research tool, is the best current database of *PKD1* and *PKD2* variants containing over 2300 variants identified in individuals with polycystic kidney disease, but novel variants are often identified. In patients with a high pretest probability of ADPKD on the basis of clinical criteria, but no finding of a pathogenic (P) or likely pathogenic (LP) variant in a cystic kidney gene, additional evaluation of cystic gene VUS can be helpful. In this case-based review, we propose an algorithm for the assessment of such variants in a clinical setting and show how some can be reassigned to a diagnostic grouping. When assessing the relevance of a VUS, we consider both patient/family-specific and allele-related factors using population and variant databases and available prediction tools, as well as genetic expertise. This analysis plus further family studies can aid in making a genetic diagnosis.

## Introduction

**Figure 1:**
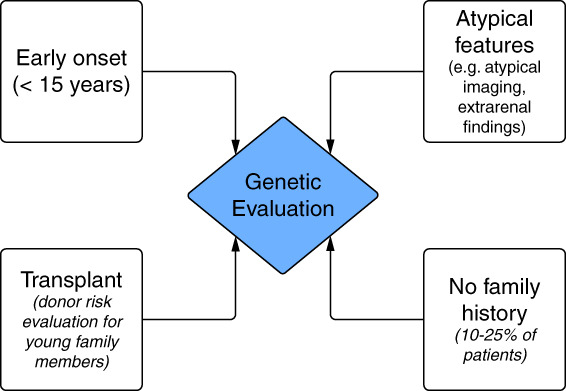
**Indications for genetic testing (expert opinion).**^2,3^ Genetic testing is useful in early-onset or very early-onset disease; in atypical imaging features, such as extrarenal findings; in donor risk evaluation for young asymptomatic individuals; and in the absence of family history.

Autosomal dominant polycystic kidney disease (ADPKD) is the most common inherited kidney disease affecting 1 in 500–1000 individuals.^1^ For patients with a family history, a diagnosis is made using the Pei criteria.^2^ However, 10%–25% of patients with ADPKD do not have a family history.^3,4^ In the absence of family history, if atypical imaging is present or if the disease course is quite different between generations, genetic testing is recommended. Genetic testing may also be indicated for determining whether a young, related potential donor has inherited the familial variant before potential kidney transplant or for consideration of *in vitro* fertilization with preimplantation genetic testing (Figure 1).^2,3^

In ADPKD cohorts enriched for rapidly progressive disease, such as HALT Progression of Polycystic Kidney Disease (HALT-PKD) study and Consortium for Radiologic Imaging Study of PKD, *PKD1* and *PKD2* variants account for approximately 78% and 15% of cases. In the remaining approximately 7%, no pathogenic variant was identified^5^. By contrast, when ADPKD was studied in a large, unselected cohort, only 77% of those with a chart-confirmed diagnosis of ADPKD had a disease-associated variant identified. Of this subset with a genetic diagnosis, 70% had rare variants in *PKD1*, 19% in *PKD2*, and 10% in other cystic kidney disease genes.^6^ Patients with mild or atypical disease were less likely to have a disease-causing variant identified. These patients had more novel variants in *PKD1* or *PKD2* or a variant in another cystic kidney disease gene (*ALG8*, *GANAB*, *IFT140*, or *PKHD1*).^6^ In a large Taiwanese ADPKD cohort study, pathogenic *PKD1* or *PKD2* variants were identified in 69% of families, with 7% due to *PKHD1*, *GANAB*, or *ALG8*.^7^

Other identified genes causing an ADPKD-like phenotype include *GANAB*, *DNAJB11*, *ALG9*, and *IFT140*.^8–12^ *COL4A1*,^13^ *COL4A3*, or *COL4A4*^14^ variants may also lead to bilateral renal cysts and kidney disease. Variants in *PRKCSH*, *SEC63*, *LRP5*, *ALG8*, *SEC61B*, and *PKHD1* have been implicated in the spectrum of autosomal dominant polycystic liver disease, but some also cause kidney cysts.^4^

The differential diagnosis for small cystic kidneys is broad, including acquired renal cystic disease, lithium-induced nephropathy, ADTKD, and *DNAJB11*-associated PKD. Given the growing recognition of the complexity of genotype–phenotype correlations in cystic kidney disease, a nomenclature to capture both the clinical diagnosis and the genotype has been proposed.^8^

*PKD1* pathogenic variants tend to result in more severe disease than *PKD2*. The variant type also affects disease progression. On average, truncating or loss-of-function variants in *PKD1* are associated with more rapidly progressing disease than missense variants in which full-length protein is synthesized.^15^ This genotypic difference along with biological sex, hypertension, or a urologic event before the age of 35 years forms the basis of the Predicting Renal Outcome in PKD (PRO-PKD) score.^16^ Genotyping does not improve risk stratification when combined with height- and age-adjusted total kidney volume (TKV)^17^; however, we offer both tools during initial discussions of risk of progression.

Shared decision making should precede any genetic testing. Patients should receive appropriate counseling before and after genetic testing.^18^

### Clinical Genetic Testing

Next-generation sequencing is a high-throughput, low-cost, high-sensitivity genetic testing method that is now widely used in clinical care.^13^ Clinical laboratories have developed gene panels tailored to specific organs (*e.g.*, a kidney gene panel) or disease patterns (*e.g.*, nephrotic syndrome). The cases we present had testing using a commercial broad targeted next-generation sequencing (tNGS) panel. The limitations of this type of testing have been previously reviewed.^18^ Although testing is optimized for the genes on the panel, a disease-associated variant may still be missed if it is not on the panel, or for other technical reasons such as when the disease-causing change is intronic or when there is mosaicism.^19^

### Reporting of Genetic Variants

The American College of Medical Genetics and Genomics (ACMG) and the Association for Molecular Pathology^14^ set guidelines for variant reporting that result in the following possible classifications: pathogenic (P), likely pathogenic (LP), variant of uncertain significance (VUS), likely benign (LB), and benign (B). LP and LB represent greater than a 90% certainty of a variant being disease causing or benign on the basis of a constellation of evidence with varying strengths, including variant frequency, functional data, co-segregation, *in silico* tools, and more. Some rare variants may be classified differently by different testing laboratories and/or some variants may be initially assigned to one class but later reclassified as further data become available. Other scoring systems have been proposed.^20,21^ In the future, a ClinGen Variation Curation Expert Panel^22^ and the broader ACMG/Association for Molecular Pathology Sequence Variant Interpretation Working Group may also provide more specific guidance on how the ACMG criteria should be applied to PKD-related genes.

## Evaluating the Phenotypic Significance of a Variant

**Figure 2:**
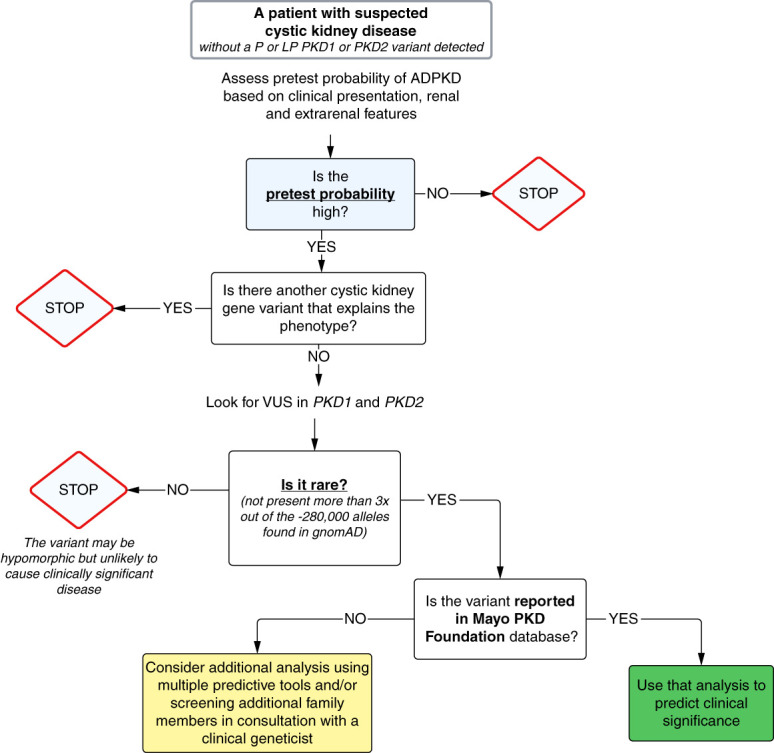
**An algorithm for assessing potential clinical relevance of a VUS in PKD.** The first step is to consider the likelihood of ADPKD on the basis of clinical features. If the pretest probability is high, check whether there are other pathogenic or likely pathogenic variants in other cystic genes. If the pretest probability is low or there is a likely pathogenic or pathogenic gene that can explain the findings, we stop further assessment. If not, we assess the variant prevalence. If the variant is present more than three times in gnomAD, then it is unlikely to cause clinically significant disease. If the variant is rare, we check the Mayo/PKDF database and other databases. If not, then we review prediction using *in silico* tools and consult with a medical geneticist/genetic counselor. ADPKD, autosomal dominant polycystic kidney disease, LP, likely pathogenic; MAF, minor allele frequency; P, pathogenic; PKD, polycystic kidney disease; VUS, variant of uncertain significance.

Like others,^6^ we found that patients with a family history of ADPKD tend to have more previously reported classified (LP or P) variants while those without a family history tend to have more variants classified as VUS. This could be because segregation is one factor supporting pathogenicity with the ACMG guidelines. We follow the algorithm in Figure 2 for further analysis. The first step is a clinical assessment of the likelihood that the patient has ADPKD on the basis of imaging and clinical features (hypertension, kidney function, and extrarenal findings). If the pretest probability is low or if the pretest probability is high but there is an additional LP or P variant in another cystic kidney gene that explains the phenotype, we do not analyze the VUS. In most cases, the VUS is a missense variant (resulting in a single amino acid change) because truncating variants are likely to be classified as disease causing. Because there is a temptation to overattribute disease causation to missense variants,^23^ ongoing collaboration with genetics experts is necessary to prevent errors.^24–26^

If our pretest probability is high for ADPKD and a VUS in a cystic kidney gene is found, then we begin by analyzing the variant prevalence. The allele frequency of a significant disease-causing variant will be rare.^27^ ADPKD is present in 1:500 to 1:1000 individuals, but because no single variant accounts for most disease, and based on our experience, we expect fully penetrant pathogenic variants to be very rare (usually not present more than 3× out of the approximately 280,000 alleles found in the large population database, the genome aggregation Database [gnomAD] v2.1.1.^28^). If minor allele frequency (MAF) is higher than this threshold, then, in our experience, the variant is unlikely to be fully penetrant. However, a more common variant (with a higher MAF) may still cause a mild phenotype with a few cysts (hypomorphic change).^29^ The report may also mention whether the variant is in ClinVar, a public archive of human variants and associated phenotypes, hosted by the National Cancer Biotechnology Institute. If a variant is not found in these databases, this also supports that it is rare.

If the variant is rare and in *PKD1* or *PKD2*, we check the Mayo/PKDF variant database,^30^ a publicly accessible, curated database funded by the PKD Foundation and maintained by the Mayo PKD Center. If the variant is not in the Mayo/PKD Foundation database, then we review commercially available algorithms. Various bioinformatics tools using supervised machine learning analysis of available human genome datasets as training datasets have been developed to assess the effect of variants.^31^ Some of these tools include MutationTaster-2,^32^ SpliceAI, Polymorphism Phenotyping-2,^31,33^ Sorting Intolerant from Tolerant,^34^ Combined Annotation-Dependent Depletion,^35^ and the more comprehensive Rare Exome Variant Ensemble Learner (REVEL)—which offers a composite of scores from 13 individual tools.^36^ These programs predict variant significance on the basis of several factors including the Grantham distance, conservation of the amino acid or nucleotide across species (the more highly conserved, the more likely it is to be disease causing), and interference with/proximity to a splice site. If a variant is rare, and the prediction tools favor pathogenicity, it will still likely score as a VUS by the reporting laboratory without segregation or some other data. We stress that caution must be used with sequence variant interpretation using these tools and encourage consultation with a genetic specialist. Given variability in performance between individual bioinformatics prediction tools, the ACMG advises against the use of a single prediction tool and recommends using a combination of tools. In the patient cases that follow, we use the more comprehensive tool REVEL,^36^ which uses a combination of tools to predict the pathogenicity of a variant.

With this approach, in the context of the clinical findings, we are sometimes able to assess whether a VUS leans toward likely pathogenic or likely benign. Additional data, such as screening both affected and unaffected family members and evaluating *in vitro* assays of the VUS, may help fully resolve the VUS. If a patient has ongoing follow-up, we periodically revisit existing databases to see whether the VUS classification has been changed. Finally, we suggest contacting the genetic testing laboratory to discuss whether a variant leans pathogenic or leans benign.

To illustrate real clinical dilemmas, we review four individuals found to have VUS in a cystic kidney disease gene. All patients provided verbal consent for sharing their information without identifiers.

## Case 1: Classic ADPKD Puzzling Family History

**Figure 3:**
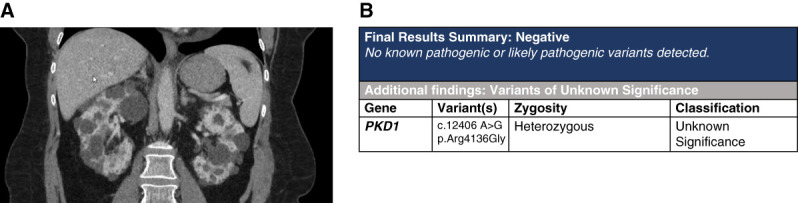
**Imaging and genetic sequencing results of case 1.** (A) Coronal CT of the abdomen with contrast. The right kidney measures approximately 18.3×10.6×7.6 cm. The left kidney measures approximately 16.9×8.7×6 cm. Both kidneys contain innumerable cysts. (B) Sequencing result report shows negative for relevant pathogenic genes and VUS in *PKD1*. CT, computed tomography.

A 56-year-old woman was diagnosed with ADPKD at 22 years when she had a symptomatic kidney stone. She now has hypertension and stage 3a CKD. Abdominal computed tomography revealed Mayo Imaging class 1C kidneys (Figure 3). She has high-risk features, including hypertension and a urologic event before the age of 35 years. She noted family members on both her maternal and paternal sides to have PKD, which would be unusual. Although she has a clinical diagnosis of typical ADPKD, a tNGS panel was ordered given the puzzling family history.

Genetic testing was negative for a disease-causing (P or LP) variant; however, she had a VUS in *PKD1*. Clinically, we had a high suspicion of ADPKD, and no other disease-causing variants explained her phenotype. Therefore, we examined the *PKD1* VUS (c.12406A>G, p.Arg4136Gly).

### Interpretation and Discussion

This variant is absent from gnomAD and present within the Mayo/PKDF database. It has a REVEL score of 0.441 and has been reported in two prior publications. Given our clinical diagnosis of ADPKD and the finding of a rare *PKD1* variant, we considered the possibility that the *PKD1* VUS was clinically important. In the Mayo/PKDF database, this variant is considered likely pathogenic (by research analysis) and was documented in another individual with ADPKD. We have not reclassified the variant but have offered the patient the interpretation as suspicious to be disease causing for her. Given her family history, we offered further genetic screening for both maternal and paternal family members to explore whether another variant is present in others.

## Case 2: Classic *De Novo* ADPKD

**Figure 4:**
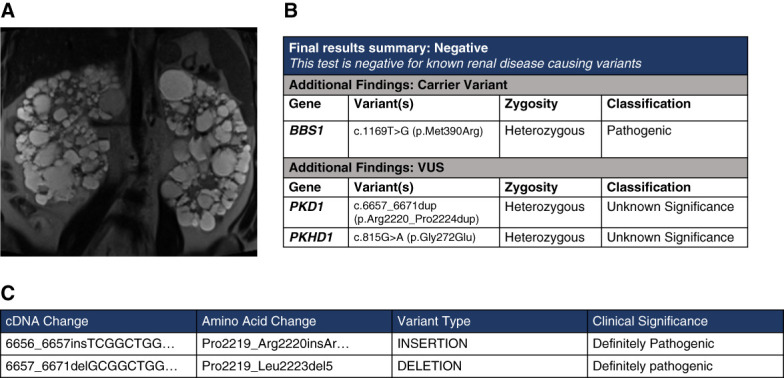
**Imaging and sequencing results of case 1.** (A) Coronal T2-weighted MRI showing large kidneys with innumerable kidney cysts. (B) The patient was negative for a pathogenic variant, but has a BBS1 carrier variant and a VUS in *PKD1* and *PKHD1*. This *PKD1* VUS is absent in gnomAD. (C) Examination of the region in the Mayo database revealed two pathogenic indels near the region. BBS, Bardet–Biedl syndrome; MRI, magnetic resonance imaging.

A 34-year-old man was known to have kidney cysts since childhood. He had a history of hypertension, uric acid nephrolithiasis, and obesity. He had no known family history of kidney disease. Abdominal magnetic resonance imaging revealed Mayo Imaging class 1E kidneys with a TKV of 4600 cc (Figure 4). He had CKD stage IIIa with serum creatinine of 1.80 mg/dl, compared with 1.6 mg/dl a year earlier. His clinical diagnosis was ADPKD with high risk of progression. Given the absence of family history, a tNGS panel was performed.

Genetic testing was negative for a disease-causing variant (P or LP). However, the patient was a carrier of a single (heterozygous) pathogenic variant in *BBS1*, a gene causing Bardet–Biedl syndrome (BBS), an autosomal recessive disease with cystic kidneys and other syndromic features.^37^ The patient also had a VUS in *PKD1*.

### Interpretation and Discussion

In this case, our pretest probability of ADPKD was very high. Although pathogenic *BBS1* variants are associated with cystic kidneys, they must be biallelic. The patient had only a single variant and does not have syndromic features of BBS other than obesity. Although it is possible the initial testing missed a second disease-causing variant of *BBS1*, clinically, we felt he had ADPKD, not BBS. We, therefore, evaluated the *PKD1* VUS.

The *PKD1* VUS, c.6657_6671dup (p.Arg2220_Pro2224dup), is predicted to result in an in-frame duplication of 15 nucleotides in *PKD1*. This variant is absent in gnomAD and had not been reported in the Human Gene Mutation Database. It is not present in the Mayo/PKDF database, but there is a pathogenic in-frame deletion at the same site in an individual with ADPKD. The variant is in exon 15, the largest exon in *PKD1*, within the PKD repeat domain. There is no *in silico* prediction available for this variant. Our assessment is that this rare *PKD1* VUS is likely clinically relevant and leans likely pathogenic. Parental testing was suggested to confirm that this was a *de novo* pathogenic variant; however, that was not possible.

This case raises the question of whether the *BBS1* variant could be contributing to the severity and early progression of disease. This *BBS1* variant (known as M390R) has been reported in 80% of homozygous and compound heterozygous patients with *BBS1* pathogenic variants.^38^ Present in 75.7% of all families with BBS1 in the United States, it is disease causing when compounded heterozygous with other *BBS* variants.^38,39^ An interaction between *BBS* genes (specifically *BBS1*) and *PKD1* has been described^40^; however, more research is needed on whether single *BBS* variants could influence cystic disease.

The patient also had a *PKHD1* VUS; however, the clinical presentation (large cystic kidneys, absence of liver cysts, and early kidney disease progression) is more consistent with the *PKD1* variant as the more likely explanation for the disease. Furthermore, this variant was estimated to be uncertain in REVEL with a score (0.3) leaning benign.

### Decisions following Genetic Testing

We felt the patient's ADPKD was likely due to the *PKD1* variant. The patient's clinical features (large TKV, hypertension, and loss of kidney function at an early age) are consistent with early progression to ESKD. Before the genetic testing, we had counseled him on BP control, diet, and lifestyle modifications and tolvaptan use. Formally, the case remains unresolved; however, genetic testing results likely support that he was at high risk of progression. This genetic information may be important for living-related donor selection and potentially family planning because a suspicious VUS may be considered for preimplantation genetic testing on a case-by-case basis.

### Take-Home Message

We suggest genetic testing for patients with typical ADPKD with a negative or unknown family history. Genetic testing results for cystic kidney disease should always include VUS data.

## Case 3: Clinical ADPKD with Exophytic Cysts

This is a 72-year-old man with PKD and a history of hemorrhagic stroke, hypertension, and stage 4 CKD. He had no known family history of kidney disease. Abdominal magnetic resonance imaging showed numerous, bilateral kidney cysts (Figure 4A). After extensive discussion, he underwent genetic testing for prognostic reasons for his children. A tNGS panel found both a LP variant and a VUS in *IFT140*, as well as a VUS in *PKD1* (Figure 4B).

### Interpretation and Discussion

The pretest probability of ADPKD was high, and the patient had a truncating *IFT140* variant. Biallelic pathogenic variants in *IFT140* cause short rib thoracic dysplasia-9.^41^ However, a strong association has been found between loss of function monoallelic variants in the *IFT140* gene and a mild, atypical bilateral cystic phenotype^9^ with autosomal dominant inheritance. The finding of multiple exophytic cysts is consistent with a monoallelic *IFT140*-related disease phenotype. In our patient's report, the variant *IFT140:* c.3250_3253dup (p.Val1085Glyfs*36) was listed as a carrier variant rather than disease causing, perhaps because the monoallelic disease has only recently been described. The *IFT140* VUS c.728A>G (p.Glu243Gly) is quite common in the Southeast Asian population (0.1% in gnomAD) and thus unlikely to be relevant. The *PKD1* VUS c.12864C>G (p.Ser4288Arg) is not rare (0.011% frequency in the South Asian population) and is predicted to be benign in REVEL.

### Decisions following Genetic Testing

The patient's cystic kidney disease presentation was attributed to the truncating *IFT140* variant rather than *PKD1* VUS. The patient and family were reassured about the lower likelihood of early progression of kidney disease with *IFT140*-associated PKD. Lifestyle modifications (BP control and diet) were advised.

### Take-Home Message

A finding of multiple exophytic cysts should trigger suspicion for either a disease-causing *IFT140* variant, the third most common genetic defect in ADPKD,^9^ or a *COL4A1* variant.^13^ Sometimes these will be reported as carrier variants. Not all cystic gene panels carry *IFT140*. Therefore, it is important to select the appropriate panel for testing.

## Case 4: The Spectrum of Cystic Disease

A 61-year-old man was referred for evaluation of kidney stones and liver cysts. He had no family history of kidney disease. He had stable and preserved eGFR and significant hypercalciuria. Ultrasound revealed multiple punctate nonobstructing renal calculi and hyperechoic medulla. An abdominal computed tomography revealed multiple small diffuse liver cysts and confirmed medullary nephrocalcinosis (Figure 6A). Genetic testing was pursued for workup of liver cysts and medullary nephrocalcinosis. A tNGS panel revealed a heterozygous pathogenic *PKHD1* variant as well as VUS in *PKD1* and VUS in *PKHD1* (Figure 6B).

**Figure 5:**
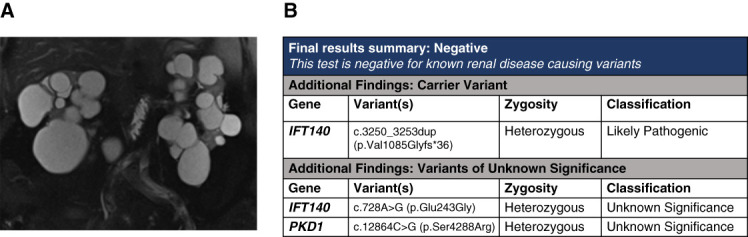
**Imaging and genetic sequencing results of case 2.** (A) Coronal T2-weighted abdominal MRI showing small kidneys with multiple cysts. (B) The patient has a carrier variant in *IFT140* and a VUS in *IFT140* and *PKD1*.

**Figure 6:**
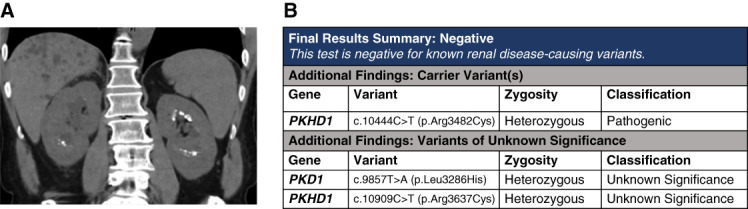
**Imaging and sequencing results of case 3.** (A) Axial CT imaging showing small, diffuse liver cysts, a few kidney cysts, and medullary nephrocalcinosis. (B) Sequencing result showing a carrier variant in *PKHD1* and VUSs in *PKD1* and *PKHD1*.

### Interpretation and Discussion

This patient does not have a classic PKD phenotype but does have a polycystic liver and medullary nephrocalcinosis. On the basis of imaging, he has a low probability of ADPKD. However, his clinical phenotype may be consistent with a heterozygous (carrier) state of *PKHD1*. The pathogenic *PKHD1* variant has been reported in a biallelic state in multiple individuals with autosomal recessive PKD (ARPKD).^42–45^ The VUS in *PKHD1* c.10909C>T (p.Arg3637Cys) is rare and has been reported in ClinVar multiple times as a VUS. A REVEL score of 0.61 further supports a VUS designation. The *PKD1* VUS is rare (MAF of 0.00002 in gnomAD) and has a low REVEL score (0.33, lean benign).

ARPKD, associated with two (biallelic) disease-causing variants of *PKHD1*, has variable penetrance with presentations ranging from embryonic demise to mild kidney and/or liver cysts in adolescence or adulthood.^46,47^ Monoallelic (carrier or heterozygous) *PKHD1* pathogenic variants are associated with atypical findings, such as increased renal echogenicity or liver predominant findings resembling polycystic liver disease.^4^ Heterozygous carriers of disease-causing *PKHD1* variants may have medullary nephrocalcinosis.^48^ The carrier rate for *PKHD1* is 1%–2% of the population, and up to 10% of adult *PKHD1* carriers have kidney cysts and multiple liver cysts.^49^ Consistent with these findings, somatic inactivation of *PKHD1* in mouse models results in biliary duct–derived liver cysts.^50^

### Decisions following Genetic Testing

The patient's clinical findings of medullary nephrocalcinosis and diffuse liver cysts and his mild kidney disease are likely correlated with the monoallelic pathogenic *PKHD1* variant.

### Take-Home Message

Mild phenotypes with kidney and liver cysts may be seen commonly in carriers of *PKHD1* disease-causing variants and should be considered in patients with medullary calcinosis and/or liver cysts.

Genetic testing is becoming more common in cystic kidney disease and is increasingly incorporated as part of an initial evaluation. We highlight that, particularly in *PKD1*, a rare, possibly disease-causing variant may be classified as a VUS by a testing laboratory. Further evaluation of the VUS may help the clinician decide whether the VUS leans toward likely pathogenic or likely benign, which may help drive the decision to test other family members and/or pursue highly specialized testing, such as functional analysis of the VUS within a center for individualized medicine. We also highlight that a heterozygous variant in *IFT140* or *PKHD1* may explain a cystic phenotype. Although *PKD1* and *PKD2* remain responsible for most ADPKD cases, other genes including those associated with autosomal dominant polycystic liver disease or ARPKD may be more common with mild or atypical phenotypes. We do not yet have enough information about these nonclassic ADPKD genotypes to use them as a basis for clinical decision making. We use height- and age-adjusted TKV for determining risk of progression in patients with preserved renal function and a clinical diagnosis of ADPKD, but these may not be reliable for non-*PKD1/PKD2* ADPKD. Clinical decisions around screening for extrarenal manifestations, such as intracranial aneurysms, should be made without regard to genetic testing results because we do not yet have enough information to limit screening in any genotype or in the absence of a genetic finding.

We strongly encourage clinicians to collaborate with genetics experts in interpretation and follow-up of a VUS particularly as knowledge and guidelines evolve.
